# Use First, Trust Later? Exploring How Health Care Providers View the Gaps Between AI’s Regulation and Its Implementation

**DOI:** 10.2196/74038

**Published:** 2025-11-10

**Authors:** Aviad Raz, Yael Inbar, Ziv Paz, Barkan Hofman, Zalman Itzhakov, Orly Weinstein

**Affiliations:** 1 Department of Sociology and Anthropology, Ben-Gurion University of the Negev, Be'er-Sheva, Be'er-Sheva, 8496500, Israel, 972 86472058; 2 Technology Management and Information Systems, Tel Aviv University, Tel-Aviv, Israel; 3 Clalit Health Services, Tel-Aviv, Israel

**Keywords:** artificial intelligence, regulation, implementation, health care management, radiology, content analysis, qualitative

## Abstract

The regulatory focus on premarket approval, overlooking and limiting the life cycle, real-world adaptiveness potential of artificial intelligence in health care, requires ongoing oversight and quality assurance by health care providers–cum-deployers, especially during early adoption.

## Introduction

This study explores how the specific nature of regulating health care artificial intelligence (AI) influences managing its deployment. Food and Drug Administration and European Commission regulations focus on premarket approval yet fail to address monitoring of AI postmarketing [1]. Better regulations for “adaptive” AI systems are needed. These are considered with precaution as “high-risk” and prohibited from fully autonomous diagnosis. As AI models are exposed to new data in clinical settings, their performance may degrade or alter over time, necessitating ongoing oversight [2,3]. This often means that health care providers must step into the regulatory ambiguity zone to develop local protocols for quality assurance during early adoption phases.

## Methods

### Overview

In-depth semistructured interviews (30‐80 min) were conducted with 65 (29 females, 36 males) managers and physicians (mainly radiologists) from 4 different Israeli hospitals. Participants, identified by hospital management, were voluntarily included as deployers of AI for medical imaging analysis. Using a grounded theory approach [4], preliminary codes were identified based on the literature review, with additional themes identified through interview analysis. The research team did the coding in parallel to ensure interrater reliability and to discuss further emerging codes. Data analysis was completed upon agreement that thematic saturation was reached.

### Ethical Considerations

This research was approved by Clalit Health Services Ethical Review Board (number 0001‐22-CNT-C). Participants signed an informed consent form; all details were anonymized to maintain privacy and confidentiality; no compensation was provided to participants.

## Results

### Overview

Many of our respondents criticized the absence of clear regulations for AI implementation, seen as challenging and at times confusing, yet also enabling experimentation.

### The Precautionary Nature of AI Regulation in Health Care and Its Postdeployment Implications

Current premarket approval processes require AI-based medical devices to stop their retraining postdeployment. This reflects precautionary regulation regarding health care AI. Our respondents expressed worries about having to become the “AI’s QA [quality assurance] testers”: “I cannot always rely on the AI in trauma cases where the injection is not good enough and it does not detect heart failure...At nights I filter the hemorrhages alerts because it is over-sensitive...I wish I could teach it, talk to it, like a real team member” (N8).

AI systems were considered “overalerting,” and managers instructed physicians to customize the alert threshold according to their needs: “We got used to all sorts of false alerts. So, we rely more on our experience and knowledge, and we combine that with the system” (M6). Managers and local “champions” (“superuser” radiologists) had to become AI supervisors: ”I make everyone here provide feedback regarding the AI system” (M5). Playing an active role in shaping the use of AI systems was nevertheless seen positively: “We feel like we are part of the development team. It feels good. It makes me even more engaged” (R1).

## Discussion

Our main study findings show how regulating health care AI influences managing its deployment by highlighting the need for customization of the AI tools that require experimentation. Strategies included vendor-appointed “champions” and on-site feedback loops [5]. Nevertheless, such customization requires further monitoring of bias metrics in model outputs and selective use by physicians.

There are some limitations to consider. Findings were drawn from interviews with radiologists, considered early adaptors of technologies, whose experiences may vary from that of other health providers. Although the study captures early adoption phases, future research should explore how this might shape long-term AI integration. In conclusion, the broader implications are that to become a sustainable approach in medical AI implementation, the “use first, trust later” mindset cannot be left unmonitored. Regulation should address adaptive AI and consider how transparency (rather than explainability) of the initial and postdeployment training, as well as bias monitoring, can enable oversight. Additionally, hospital administrators should work with AI developers to monitor biases in customization [6], as well as manage effective teaming routines with AI.

## Abbreviations

AI: artificial intelligence
